# Unlocking the Role of a Genital Herpesvirus, Otarine Herpesvirus 1, in California Sea Lion Cervical Cancer

**DOI:** 10.3390/ani11020491

**Published:** 2021-02-13

**Authors:** Alissa C. Deming, James F. X. Wellehan, Kathleen M. Colegrove, Ailsa Hall, Jennifer Luff, Linda Lowenstine, Pádraig Duignan, Galaxia Cortés-Hinojosa, Frances M. D. Gulland

**Affiliations:** 1 The Pacific Mammal Center, Laguna Beach, CA 92651, USA; 2 Aquatic Animal Health and Comparative, Diagnostic and Population Medicine, College of Veterinary Medicine, University of Florida, Gainesville, FL 32608, USA; wellehanj@ufl.edu (J.F.X.W.); galaxia.cortes@uc.cl (G.C.-H.); 3 Veterinary Sciences, The Marine Mammal Center, Sausalito, CA 94965, USA; duignanp@tmmc.org (P.D.); francesgulland@gmail.com (F.M.D.G.); 4 Zoological Pathology Program, College of Veterinary Medicine, University of Illinois at Urbana-Champaign, Brookfield, IL 60513, USA; kcolegro@illinois.edu; 5 Sea Mammal Research Unit, Scottish Oceans Institute, School of Biology, University of St. Andrews, St. Andrews KY16 9AJ, UK; ajh7@st-andrews.ac.uk; 6 Population Health and Pathobiology, College of Veterinary Medicine, North Carolina State University, Raleigh, NC 27606, USA; jaluff@ncsu.edu; 7 Pathology, Microbiology and Immunology and Karen C. Drayer Wildlife Health Center, School of Veterinary Medicine, University of California Davis, Davis, CA 95616, USA; ljlowenstine@ucdavis.edu; 8 Current address: School of Veterinary Medicine, Pontificia Universidad Católica de Chile, Avenida Vicuña Mackenna 4860, Santiago 7820436, Chile

**Keywords:** California sea lion, cancer, urogenital carcinoma, herpesvirus, oncogenic virus, RNA in situ hybridization, Basescope^®^

## Abstract

**Simple Summary:**

Wild California sea lions (*Zalophus californianus*) have a high prevalence of urogenital carcinoma. The cancer starts in the sea lion’s genital tract then spreads aggressively to other organs resulting in death. Previous research has identified a herpesvirus, otarine herpesvirus 1 (OtHV1), in the genital tract of most sea lions with urogenital carcinoma, however, this virus has also been found in the genital tracts of sea lions without cancer making its role in urogenital carcinoma ambiguous. Here, tissues from 95 sea lions with and 163 without cancer were tested for OtHV1, the amount of virus was quantified, and viral gene expression was measured. OtHV1 was found in 100% of the sea lions with urogenital carcinoma and there were exceptionally high viral loads and viral gene expression within the genital tumors. Of the sea lions that did not have cancer, 36% tested positive for herpesvirus and they had much lower viral load and no detectable viral gene expression, indicating the herpesvirus was dormant. These findings support that genital herpesvirus plays an integral role in sea lion urogenital carcinoma and suggests there is an underlying trigger or event that causes the virus to induce cancer in some infected sea lions and not others.

**Abstract:**

Urogenital carcinoma in California sea lions (*Zalophus californianus*) is the most common cancer of marine mammals. Primary tumors occur in the cervix, vagina, penis, or prepuce and aggressively metastasize resulting in death. This cancer has been strongly associated with a sexually transmitted herpesvirus, otarine herpesvirus 1 (OtHV1), but the virus has been detected in genital tracts of sea lions without cancer and a causative link has not been established. To determine if OtHV1 has a role in causing urogenital carcinoma we sequenced the viral genome, quantified viral load from cervical tissue from sea lions with (*n* = 95) and without (*n* = 163) urogenital carcinoma, and measured viral mRNA expression using in situ mRNA hybridization (Basescope^®^) to quantify and identify the location of OtHV1 mRNA expression. Of the 95 sea lions diagnosed with urogenital carcinoma, 100% were qPCR positive for OtHV1, and 36% of the sea lions with a normal cervix were positive for the virus. The non-cancer OtHV1 positive cases had significantly lower viral loads in their cervix compared to the cervices from sea lions with urogenital carcinoma. The OtHV1 genome had several genes similar to the known oncogenes, and RNA in situ hybridization demonstrated high OtHV1 mRNA expression within the carcinoma lesions but not in normal cervical epithelium. The high viral loads, high mRNA expression of OtHV1 in the cervical tumors, and the presence of suspected OtHV1 oncogenes support the hypothesis that OtHV1 plays a significant role in the development of sea lion urogenital carcinoma.

## 1. Introduction

Wild California sea lions (*Zalophus californianus*) have the highest prevalence of cancer among the marine mammal species [1,2]. Over the last three decades, postmortem examinations of adult sea lions that stranded along the central and northern coastline of California, USA concluded that approximately one in four had cancer [1,2,3]. The vast majority (>90%) of these cancers were of the same type, urogenital carcinoma [2]. This cancer originates in the genital tract (cervix, vagina, penis, or prepuce) and aggressively spreads to the draining lymph nodes, liver, lungs, spleen, and/or kidneys, resulting in death [1,2,4,5]. There have been several studies of the etiology of this urogenital carcinoma, but much remains uncertain about its cause.

Even in well-studied species, like humans or domestic animals, identifying the etiology and cofactors contributing to the development of cancer is difficult. This is especially true in wild animals with limited accessibility and clinical history. This is because most cancers result from a multistep process and can involve several contributing factors in the progression of cellular transformation to malignancy. These can include a combination of immune suppression, genetic predisposition, toxin exposure, and/or infectious agents in addition to random accumulation of genetic mutations [6].

In previous studies, some of these potential cofactors have been epidemiologically associated with California sea lion urogenital carcinoma. For example: legacy contaminants including polychlorinated biphenyls (PCBs) and dichlorodiphenyltrichloroethane (DDT) are eight and six times higher, respectively, in blubber of sea lions with urogenital carcinoma [7,8]; sea lions with cancer were found to be more inbred than those dying from other causes and are more likely to be of certain genotypes [9,10]; and a herpesvirus infection, initially named California sea lion herpesvirus (GenBank accession # AF193617.1) and more recently referred to as otarine herpesvirus 1 (OtHV1), has been detected more often in the genital tract of sea lions with urogenital carcinoma than in those without this cancer [4,5,11,12]. Here we look further into the viral component of this cancer to determine if OtHV1 plays an integral role in the pathogenesis of urogenital carcinoma, or if it is an incidental finding of a virus with high penetrance in this population.

Viral infections contribute to the development of 15 to 20% of cancers in humans [13,14,15]. Viruses that have the potential to cause cancer are known as oncogenic viruses and include both RNA (Retroviridae and Flaviviridae) and DNA virus families (Herpesviridae, Papillomaviridae, Hepadnaviridae, Adenoviridae, Poxviridae, and Polyomaviridae) [13,15,16,17,18,19]. Oncogenic viruses associated with malignancies in humans include: human gammaherpesvirus 4 (HHV4, aka Epstein-Barr virus) associated with Burkitt lymphoma, Hodgkin’s disease, nasopharyngeal carcinoma, gastric adenocarcinomas, and post-transplant lymphomas; human gammaherpesvirus 8 (HHV8, aka Kaposi’s sarcoma-associated herpesvirus) associated with Kaposi’s sarcoma, multicentric Castleman’s disease, and primary effusive lymphoma; human papillomaviruses (HPV) associated with cervical cancer, anogenital cancer, skin cancer, and head and neck cancers; hepatitis B and C associated with hepatocellular carcinoma; and human T-lymphotropic virus 1 (HTLV-1) associated with the adult T-cell leukemia. In non-human species, similar oncogenic viruses are associated with neoplasms, documented in a wide range of species from primates to chickens and frogs [18,20,21]. Across the viral families and in different host species, the host cellular pathways targeted by oncogenic viruses are often analogous [19,22]. These highly conserved cellular pathways control fundamental processes including cellular proliferation, apoptosis, immune modulation, epigenetic modifications, and signal transduction pathways [23]. In the case of oncogenic viruses, this manipulation often results in increased cellular and viral replication, interference with apoptotic pathways, and enables virally infected cells to evade immune surveillance—all pushing the cell down a path of malignancy [6,16,19].

Often the progression from viral infection to virally induced cancer is associated with cofactors that contribute to whether or not an infected cell becomes neoplastic. For example, in humans close to 100% of adults carry one or more of eight types of endemic human herpesviruses [15,24,25,26]. Two of these, human HHV4 and HHV8, are known to induce cancers, but this malignant transformation only occurs in a very small subset of infected people [19,27,28,29]. This neoplastic transformation is often attributed to cofactors such as immunosuppression or toxin exposure [29,30,31]. For example, AIDS patients with human herpesvirus 8 (HHV8) infection are 500 times more likely to develop Kaposi’s sarcoma compared to the general population [29]. The high penetrance of these viruses combined with the influences of various cofactors make it challenging to establish a causal relationship between a viral infection and the development of cancer when only assessing the infection status.

After initial infection, herpesviruses typically become latent in their endemic host and rarely cause significant clinical disease [32]. However, viral recrudescence or latent herpesvirus proteins can induce disease in a subset of infected individuals, often associated with the cofactors above. Expression patterns of key viral genes (viral oncogenes) and non-coding RNAs have been implicated in determining whether a herpesvirus remains latent, benign, and subclinical, or results in cellular transformation sending the host cell down a path of malignancy. Thus, in addition to determining if a host is infected with an oncogenic virus, it is imperative to assess the latent/lytic state of the virus and the pattern of viral oncogene expression when trying to determine if a virus is responsible for inducing cancer.

Because of ethical concerns and federal laws protecting the marine mammals in the USA, proving Koch’s postulates to determine the role of OtHV1 in sea lion carcinoma by infecting sea lions with OtHV1, observing the development of this cancer, and re-isolating the virus from tumor tissue is not feasible. Instead, molecular techniques and epidemiologic approaches must be used [33,34]. Here, we tested sea lion cervix for OtHV1 infection, then measured the viral loads using quantitative PCR (qPCR) to determine if the virus was dormant or actively being expressed in infected individuals. Infection status and viral loads were then compared between cancer and non-cancer cases. Next, a draft genome of OtHV1 was assembled and annotated. OtHV1 genes were compared to known oncogenic herpesviruses to identify the potential viral oncogenes in OtHV1. Last, RNA in situ hybridization (RISH) probes were designed to visualize the OtHV1 mRNA expression within urogenital carcinoma lesions as well as in normal cervix in sea lions that tested positive for the virus. By assessing these parameters in sea lions with and without urogenital carcinoma, we can determine whether OtHV1 plays a significant role in the pathogenesis of urogenital carcinoma or is simply an incidental finding.

## 2. Materials and Methods

### 2.1. Sample Collection

California sea lions in this study stranded along the central and northern California coast from 2002 to 2017 and were admitted to The Marine Mammal Center in Sausalito, CA, USA for medical care (Marine Mammal Protection Act permit number 18,786). Because of the advanced disease, the sea lions in this study died naturally while undergoing treatment or were humanely euthanized. All sea lions included in this study were adult females. Complete necropsy and histologic evaluation were performed to establish the cause of death, determine the presence/absence of urogenital carcinoma, and cervix samples were collected and frozen (−80 °C) for OtHV1 molecular analysis [35].

Samples of normal cervix or cervical tumors when grossly discernable were collected. Cervix samples were fixed in 10% neutral buffered formalin (NBF), embedded in paraffin, sectioned at 5-µm thickness, stained with hematoxylin and eosin (H&E), and evaluated by a pathologist with expertise in sea lion urogenital carcinoma. Each case was classified as urogenital carcinoma (cancer cases) or non-urogenital carcinoma (control cases) based on histologic evaluation as previously described [36,37]. Briefly, sea lions were classified as control cases if there was normal cervical epithelium composed of pseudostratified columnar epithelium and no evidence of urogenital carcinoma on gross or histological evaluation (Figure S1) [37]. Sea lions with urogenital carcinoma had evidence of moderate to markedly dysplastic cervical epithelium with atypical parabasal cell proliferation extending from one-third to the entire thickness of the epithelium (Figure S2) and/or invasive cervical lesions (Figure S3) with or without evidence of metastasis were classified as cancer cases.

The cervix samples used for RNA in situ hybridization (*n* = 25) were collected between 5 min and 24 h postmortem and immediately fixed in 10% NBF for no more than 36 h (*n* = 11) or frozen (−80 °C) then fixed in 10% NBF for 24 h (*n* = 14) before being processed as described above. On histologic evaluation, lesions in this subset of cases were further classified into either cervical intraepithelial lesions (CIN) (Figure S2) or invasive lesions (Figure S3) [36] which approximate those in an established grading scale used in human cervical and vaginal neoplasms [38].

A second cervical tissue sample from the same area sampled above was frozen (−80 °C) until processed for OtHV1 qPCR analysis and OtHV1 genomic sequencing. Illumina MiSeq, Pacific Biosciences (PacBio) single molecule real time (SMRT), and Sanger sequencing platforms were used to obtain the OtHV1 genome from the cervical tumor DNA extract (described below). The entire MiSeq and PacBio runs were dedicated to total DNA extracted from this single tumor sample to minimize the herpesvirus strain variability.

Otarine herpesvirus 4 (OtHV4), a closely related herpesvirus identified in the genital tract of northern fur seals, was also sequenced and compared to OtHV1. Urogenital carcinoma has not been documented in the northern fur seals, and to date, no disease progression has been identified in association with OtHV4 infection [39]. For OtHV4 genome sequencing, a vaginal swab collected from a northern fur seal (*Callorhinus ursinus*) on the Pribilof Islands, Alaska, USA (Marine Mammal Protection Act permit number 932-1905iMA-009526), previously identified to be positive for OtHV4 with a high viral load, was used [39]. This was a wild fur seal sampled during routine health assessments of the population and was determined to be healthy.

### 2.2. PCR and qPCR

DNA was isolated from all California sea lion cervix biopsies using a Qiagen kit according to the manufacturer’s instructions (DNeasy Blood and Tissue, Qiagen Inc., Valencia, CA, USA). For the northern fur seal vaginal swab, DNA was isolated using a Maxwell automated extractor and Maxwell 16 Buccal Swab Purification kit according to the manufacturer’s instructions (Promega, Madison, WI, USA). PCR, qPCR, and/or viral genome sequencing was performed immediately, or DNA extracts were stored at −80 °C pending analysis.

For OtHV1, novel PCR and qPCR primers and probe were designed (Table 1) to target unique areas of the OtHV1 DNA-dependent DNA polymerase gene with Black Hole Quencher probe and FAM reporter dye and validation, sensitivity and specificity of these primer sets were performed (Supplemental Material Methods S1). DNA extracts from California sea lion cervix samples (*n* = 258) were screened for OtHV1 using a 7500 Fast Real-Time PCR System (Applied Biosystems) with OtHV1-specific qPCR primers, probe, and conditions described in the supplemental material (Methods S1).

To determine if there was a difference between the proportion of sea lions in the cancer and control groups that were infected with OtHV1, a binomial ANOVA was used. Additionally, descriptive statistics, quartile values, and a Mann–Whitney test were used to determine if there were higher viral loads (copies/ng DNA) in cervical tissue with urogenital carcinoma compared to the normal cervix that was positive for OtHV1. Lastly, a binomial general linear model was used to determine the difference in OtHV1 abundance (viral load) between cancer cases and non-cancer cases. Goodness of fit was assessed and there were no outliers identified.

### 2.3. Viral Genome Sequencing

One California sea lion cervical tumor, with a high viral load on qPCR results, was selected for OtHV1 genome sequencing. This sample was PCR and qPCR negative for OtHV4 and positive for OtHV1, with approximately 1.7 × 10^7^ viral copies detected per nanogram of DNA. One northern fur seal vaginal swab was selected for OtHV4 genome sequencing. This sample was PCR and qPCR negative for OtHV1 and positive for OtHV4, with a viral load indicated by qPCR of approximately 3.44 × 10^5^ viral copies detected per nanogram of DNA.

Illumina MiSeq, Pacific Biosciences (PacBio) single molecule real time (SMRT) and Sanger sequencing platforms were used to obtain the OtHV1 genome from the cervical tumor DNA extract. The entire MiSeq and PacBio runs were dedicated to total DNA extracted from this single tumor sample to minimize the herpesvirus strain variability and facilitate viral genome assembly.

The DNA library for OtHV1 was constructed using a Nextera XT sample preparation kit as directed by the manufacturer (Illumina). The library was quantified using a Qubit 2.0 spectrophotometer (Life Technologies, Carlsbad, CA, USA) and analyzed for size distribution using a Bioanalyzer (Agilent). Sequencing was performed on an Illumina MiSeq with 2 × 300-nt paired-end reads following standard Illumina protocols. Preliminary bioinformatics analysis (see methods below) produced a ~150 Kbp draft genome for OtHV1 composed of three large non-overlapping contiguous sequences (contigs). Further attempts to bridge the gaps in the MiSeq generated OtHV1 contigs were performed using PacBio SMRT and conventional PCR as described in the Supplemental Materials (Methods S2).

Next, Illumina MiSeq and Sanger sequencing platforms were used to sequence the OtHV4 genome from total DNA extracted from a northern fur seal vaginal swab. For the MiSeq run, a DNA library was constructed using Nextera XT sample preparation kit as directed by the manufacturer (Illumina). The library was quantified using a Qubit 2.0 spectrophotometer (Life Technologies, Carlsbad, CA, USA) and analyzed for size distribution using a Bioanalyzer (Agilent). The library was then sequenced on an Illumina MiSeq with 2 × 300-nt paired-end reads following standard Illumina protocols. Additional OtHV4 gap closure was attempted using novel primers (design based on generated draft OtHV4 genome) for conventional PCR using platinum Taq DNA polymerase (Invitrogen, Carlsbad, CA, USA), GC RICH PCR System (Sigma-Aldrich, Atlanta, GA, USA), and TaKaRa Ex Taq^®^ Hot Start Version (Takara Bio, Mountain View, CA, USA).

Sequence data for both OtHV1 and OtHV4 MiSeq runs were stored on BaseSpace and transferred into CLC Genomics 9.5.3 for trimming, genome assembly, and annotation. To generate the OtHV1 genome, a de novo assembly was performed on trimmed reads using CLC Genomics with default settings. Contigs larger than 2 Kbp were selected and a BLAST search for similarity with known herpesviruses in the nucleotide collection (nr/nt) database was performed to identify the viral contigs from sea lion genomic DNA. OtHV1 contigs were ordered for display purposes similar to HHV8 (GenBank number: NC_009333.1). The PacBio reads were then assembled using the MiSeq OtHV1 draft genome (three large contigs) as scaffolding in an attempt to fill the gaps and confirm the MiSeq-generated OtHV1 draft genome assembly. For OtHV4, de novo assembly was performed using the same method described above. In addition, the northern fur seal MiSeq paired-end reads were assembled using the OtHV1 draft genome as a scaffold.

To predict putative protein coding genes, open reading frames (ORFs) with a start codon of AUG that were larger than 150aa were identified using CLC Genomics. Additional ORFs larger than 100aa were also explored, but only included when associated with an *E*-value of <0.01 on a BLASTP search. These ORFs were then searched against the pfam database and Genome Annotation Transfer Utility (GATU, http://athena.bioc.uvic.ca/virology-ca-tools/gatu/ accessed on 3 February 2021) and named according to their associated homologues when available. Additional ORFs were identified using BLAST searches, and a small number did not have a known function and/or predictable putative protein. These unidentified ORFs were annotated with a prefix of “Ot,” for Otarine, and a number that corresponded to its novel gene order starting at the 5-prime end of the genome.

### 2.4. Viral Phylogenetic Analysis

Predicted amino acid sequences of four herpesvirus core genes (polymerase, terminase, glycoprotein B, and major capsid protein) from OtHV1, OtHV4, and twenty-seven herpesviruses representative of all known herpesvirus genera were used for viral phylogenetic analysis (Tables S1 and S2). Homologous sequences were aligned using MAFFT (https://www.ebi.ac.uk/Tools/msa/mafft accessed on 3 February 2021) [27]. Bayesian analyses of each alignment were conducted using Mr. Bayes 3.2.6 on the CIPRES server with mixed amino acid substitution models, gamma distributed rate variation, and a proportion of invariable sites [26,28]. A total of four chains were run, and statistical convergence was assessed via the average standard deviation of split frequencies and potential scale reduction factors of the parameters. Human alphaherpesvirus 3 (GenBank Accession # NC_001348) was selected as the outgroup for the core gene analyses. The initial 25% of 2,000,000 iterations were discarded as burn in. When no significant differences in topology were seen in the core gene analyses, the core gene alignments were concatenated, and the concatenated alignment was used for Bayesian analysis using the same methodology.

Maximum Likelihood (ML) analyses of each alignment were performed using RAxML-HPC2 version 8.0.24 on the CIPRES server [29] with a gamma distributed rate variation and proportion of invariable sites. Human alphaherpesvirus 3 (GenBank Accession # NC_001348) was selected as the outgroup for the core gene analyses. Bootstrap analysis was used to test the strength of the tree topology; 1000 resamplings were performed [30]. When no significant differences in topology were seen in the core gene analyses, the core gene alignments were concatenated, and the concatenated alignment was used for maximum likelihood analysis using the same methodology.

### 2.5. RNA In Situ Hybridization

To localize OtHV1 mRNA expression in urogenital carcinoma, five RISH probes (Basescope^®^, Advanced Cell Diagnostics Inc., Newark, CA, USA) were designed to specifically bind to OtHV1 genes that were thought to have oncogenic potential, including vBCL2, vFLIP, vCDK4, vEVE, and EBNA1-like (Table S3). Additionally, a positive control designed to bind to sea lion housekeeping gene DNA-dependent RNA polymerase II (dpolR2A) and a negative control dihydrodipicolinate reductase (dapB) of *Bacillus subtilis* were used for quality control. To optimize specificity, we utilized the Basescope^®^ platform to ensure the host-derived OtHV1 gene would not cross react with homologous host cellular genes.

Visualization of five OtHV1 gene transcripts was performed using custom Basescope^®^ probes and BasescopeTM^®^ Red Reagent Kit (Advanced Cell Diagnostics (ACD), Hayward, California, USA, cat #322910) following the manufacturer’s protocol with minor modification (Supplemental Materials Methods S3). Following IHC staining, each tissue section was imaged using CellSens Entry 1.11 software (Olympus) slide scanner on an Olympus BX46 with a 40X objective (area in field of view = 0.34 mm^2^). Images were imported into ImageJ. Regions of interest were defined as normal epithelium in controls or neoplastic epithelium in cancer cases. Using the ImageJ polygon tool (Supplemental Materials Methods S4), representative areas of normal cervix, CIN, and invasive urogenital carcinoma lesions were selected, avoiding regions of inflammation, necrosis, underlying stroma, or poor-quality staining—for instance, at the cut edge of the sections. The software quantified the area of the positive hybridization signal (representing any positive stained pink dot) as well as the unstained area in the region selected. The total positive hybridization signal area was divided by the total area of interest and defined as the percent positive hybridization signal.

The percent positive hybridization was compared between healthy, CIN, and invasive samples for all RISH probes as well as the positive and negative controls. Descriptive tables (quartiles) were generated from the percent positive hybridization signal to determine the extent of probe binding for healthy, CIN, and invasive samples. To compare the proportion of positive hybridization signal in non-cancer animals to CIN and invasive lesions in cancer animals, a generalized linear mixed model with a binomial distribution and a log-odds (logit) link function was used, as data were not normally distributed. A Tukey test was used to investigate the differences between groups (Healthy, CIN, and Infiltrative) in order to determine the significance, with a *p*-value of <0.01. Descriptive statistics (minimum, first quartile, median, second quartile, and maximum) for percent positive hybridization signal were generated for all OtHV1 probes, as well as negative and positive probe controls.

## 3. Results

### 3.1. Histologic Determination of Urogenital Carcinoma and Control Cases

From 2002 to 2017, cervix samples were collected at necropsy from 258 sea lions. Histologic evaluation showed 63% (*n* = 163) of the sea lions had normal cervical epithelium with no evidence of urogenital carcinoma and 37% (*n* = 95) had urogenital carcinoma (Tables S4 and S5). Within the subset of cases used for RNA in situ hybridization (*n* = 25), 9 had a normal cervix and 16 had carcinoma. The cervical lesions of the carcinoma cases were further classified histologically as CIN (*n* = 7) or invasive (*n* = 9) lesions (Table S5).

### 3.2. PCR and qPCR

#### 3.2.1. OtHV1 PCR and qPCR Validation

All 10 samples from known OtHV1 positive California sea lion cervical tumors were positive with the novel OtHV1-specific PCR and qPCR primers confirmed to be OtHV1 with Sanger sequencing; 10 OtHV1 negative California sea lion cervix samples were all negative; and all non-OtHV1 herpesvirus positive samples (OtHV4, OtHV3, and sea turtle herpesvirus) were negative using the novel OtHV1-specific PCR and qPCR primer sets. To confirm the lack of cross reactivity between OtHV1 and OtHV4, all above mentioned samples were also tested for OtHV4 using PCR and qPCR with previously published primers, probe, and conditions [39]. All samples tested negative for OtHV4 except the four known OtHV4 positive vaginal swabs from the northern fur seals.

#### 3.2.2. OtHV1 qPCR Testing and Viral Quantification

Frozen subsections of the cervix were used for viral quantification (viral copies per nanogram of DNA on qPCR) in control versus cancer samples (cervix and cervical lesions, respectively). All cervical samples (control = 163; cancer = 95) were tested using the OtHV1-specific qPCR. OtHV1 was detected in 100% (95/95) of cancer cases compared to 36% (59/163) of control cases. The proportion of cancer cases that were positive for OtHV1 was significantly different (*p* < 0.0001) from the number of control cases that were positive for OtHV1.

**Figure 1:**
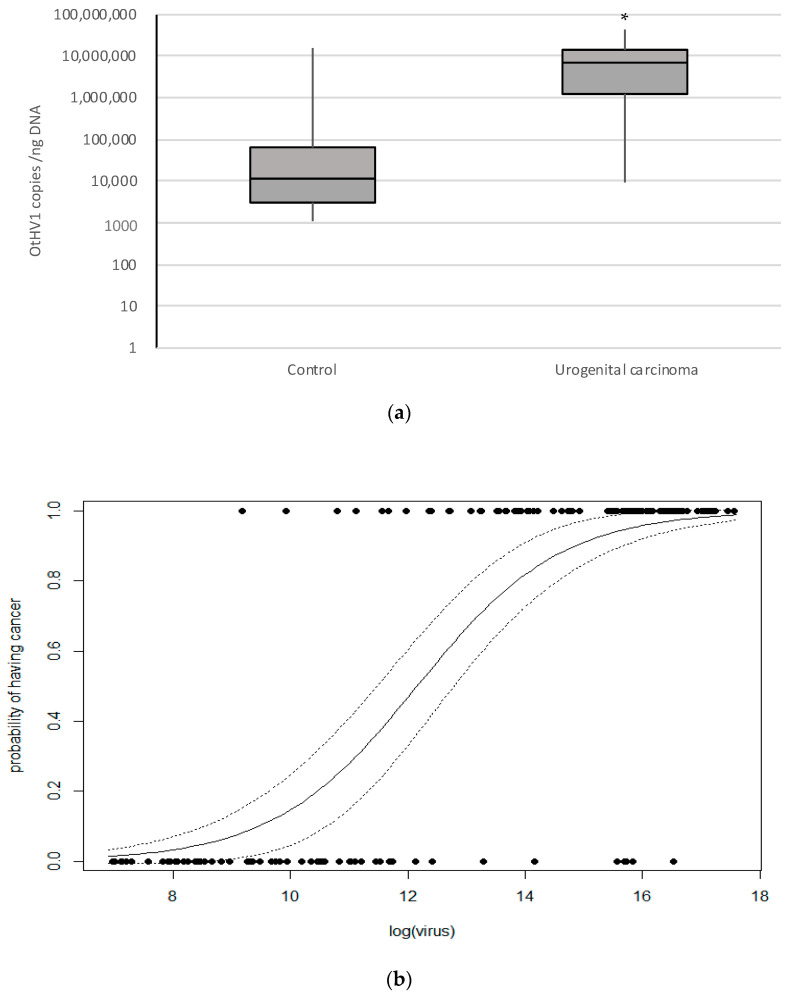
The above graphs show OtHV1 qPCR results from California sea lion cancer and control cases that were qPCR positive for OtHV1. (**a**) Box plot representing OtHV1 viral copies per nanogram of DNA detected (copies/ng DNA) determined by OtHV1 qPCR, including all OtHV1 positive histologically normal cervix (control; *n* = 59) and urogenital carcinoma (cancer; *n* = 95) cases. * = significantly different (*p* < 0.0001). (**b**) Binomial general linear model of the probability of having cancer based on the abundance of OtHV1 (log of number of viral copies detected per nanogram of DNA in the reaction) quantified with OtHV1-specific qPCR. Non-cancer cases are on 0.0 and cancer case are on the 1.0 lines.

Viral load of OtHV1 positive control (*n* = 59) and cancer (*n* = 95) cases were compared (Table S6). In normal cervix that was OtHV1 positive the median viral load was 11,551, and in urogenital carcinoma cases the median viral load was 6,704,760 (Table S6). Viral loads in control and cancer cases were not normally distributed, and non-parametric analysis was used to determine the significance. The Mann–Whitney test showed an extremely statistically significant difference (*p* < 0.0001) in viral load between the control group (*n* = 59, median = 11,551 copies/ng DNA) and cancer group (*n* = 95, median = 6,704,760 copies/ng DNA) (Figure 1A; Table S6). This proved the viral load was significantly lower in the cervical tissue of controls that were qPCR positive for OtHV1 compared to the viral load in sea lion cervix with urogenital carcinoma.

Using these OtHV1 viral loads produced from qPCR on cervix, a binomial general linear model of the probability of having cancer based on the abundance of OtHV1 (viral copies/ng DNA) showed that higher viral loads increase the probability of having urogenital carcinoma in OtHV1 positive cervical biopsies (Figure 1B). This model can be used to interpret the significance of an OtHV1 positive cervical biopsy by predicting the probability of having cancer given the abundance of the virus (viral copies/ng DNA) in an animal with unknown cancer status. By screening the cervical biopsies for urogenital carcinoma with OtHV1 qPCR in addition to the histologic evaluation, clinicians can use this model to predict the probability of an OtHV1-positive sea lion to have cancer based on OtHV1 viral load.

### 3.3. OtHV1 and OtHV4 Sequencing and Annotation

To further understand the role of OtHV1 in urogenital carcinoma, its genome was sequenced, annotated, and evaluated to identify the potential viral oncogenes. De novo assembly of the Illumina MiSeq data generated an OtHV1 draft genome consisting of three non-overlapping herpesvirus contigs. Neither the PacBio SMRT nor conventional PCR bridged the gaps between the three OtHV1 contigs generated by the MiSeq run de novo assembly, and these contigs could not be joined with bioinformatic manipulation or walking PCR. Thus, the draft OtHV1 genome consists of three contigs: an approximately 41 Kbp contig ranging from the 5′ end to ORF11 (Contig1: MN334559), a 93 Kbp contig ranging from ORF17 to ORF69 (Contig2: MN334560), and a 17 Kbp contig ranging from ORF75 to the 3′end (Contig3: MN334561) (Figure S4B). For display purposes, contigs were ordered similar to HHV8 (GenBank number: NC_009333.1) (Figure S4A).

A related pinniped herpesvirus, OtHV4, was also sequenced from a northern fur seal vaginal swab for comparison. This sample had much lower OtHV4 viral loads, and the majority of sequence obtained was host genome. Similar viral genome sequencing and assembly limitations were seen with OtHV4 that occurred with the OtHV1 genome. There were three gaps in the OtHV4 genome, resulting in four non-overlapping contigs (GenBank numbers: Contig1 = MN545486, Contig2 = MN545487, Contig3 = MN545488, Contig4 = MN545489) (Figure S4C). The gene order of OtHV4 was similar to OtHV1 and HHV8 and the contigs were ordered similarly for display purposes. Two of the gaps in OtHV4 were in the same areas as the gaps in OtHV1 (between ORF11 and ORF 17; and between ORF 69 and ORF 75). In HHV8, these areas of the genome have high GC content and tandem repeats, which may explain the difficulty sequencing and assembling these areas in OtHV1. This is compounded by the high concentration of host DNA in the samples during sequencing. Additionally, viral integration into the host genome cannot be ruled out and may contribute to the difficulty closing the OtHV1 and OtHV4 genomes when employing these sequencing and assembly techniques.

Annotation of the OtHV1 genome identified several genes similar to known gammaherpesvirus oncogenes, including Epstein-Barr Nuclear Antigen 1 (EBNA1), viral Fas-associated death domain-like interleukin-1β-converting enzyme-inhibitory protein (vFLIP), viral B-cell lymphoma 2 (vBCL2), viral cyclin-dependent kinase 4 (vCDK4), and a suspected endogenous viral elements (vEVE). In human herpesvirus-infected cells, EBNA1 and vFLIP are viral promoters and influence the host cellular pathways involved in cell proliferation including apoptosis, immune modulation, epigenetic modification, and signal transduction pathways [23,40]. vBCL2 interferes with apoptotic pathways and blocks autophagy, which in turn helps virally infected cells evade immune surveillance [41]. Viral cyclins are transcriptional regulators that help initiate host cell cycling [42]. Lastly, EVEs are viral elements inserted into either host and viral genomes that are thought to be derived from retroviruses, and in some instances have been associated with malignancies [15]. Transcription of these potential oncogenes in California sea lion urogenital carcinoma and normal cervical epithelium were further investigated using RISH.

### 3.4. Phylogenetic Analysis

**Figure 2:**
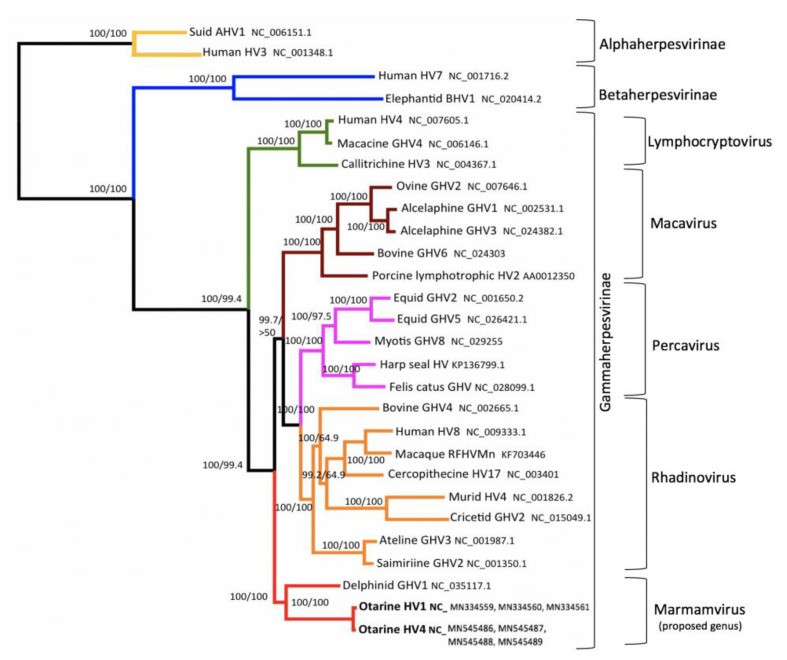
Bayesian analysis phylogram based on 4 concatenated herpesvirus core genes (polymerase, terminase, glycoprotein B, and major capsid protein) using predicted amino acid sequences. Bayesian posterior probabilities of each branch are expressed as % (**top**) and maximum likelihood (ML) bootstrap values as % (**bottom**). The 100%/100% values. Branch color is based on grouping herpesvirus subfamilies (yellow-Alphaherpesvirinae, blue-Betaherpesvirinae) and genera (green-lymphocryptoviruses, maroon-macaviruses, pink-percaviruses, orange-rhadinoviruses, and red- proposed genus Marmamvirus). Otarine HV1, Otarine HV4 and Delphinid GHV1 branch form a clade distinct from other known gammaherpesvirus genera.

Concatenated Bayesian and maximum likelihood phylogenetic analyses using four herpesvirus core genes (polymerase, terminase, glycoprotein B, and major capsid protein) from a diverse representation of herpesviruses (Tables S1 and S2) were performed. There was an early branching of the otarine herpesviruses (OtHV1 and OtHV4) within the gammaherpesvirus subfamily, divergent to a degree consistent with a new genus of herpesviruses that includes OtHV1 and OtHV4 and a herpesvirus sequenced from a proliferative lesion on the genital epithelium of a wild common bottlenose dolphin (Delphinid gammaherpesvirus 1; GenBank number: NC_035117.1) [43]. It appears that this clade branched from other gammaherpesviruses early in the subfamily’s evolution; the lack of terrestrial mammal representatives to date is interesting (Figure 2). A taxonomic proposal has been submitted to the International Committee on Taxonomy of Viruses supporting the classification of these viruses into a new genus within the family Herpesviridae (TaxoProp Marmaherpesvirus).

### 3.5. OtHV1 mRNA Expression in Urogenital Carcinoma and Control Cases

**Figure 3:**
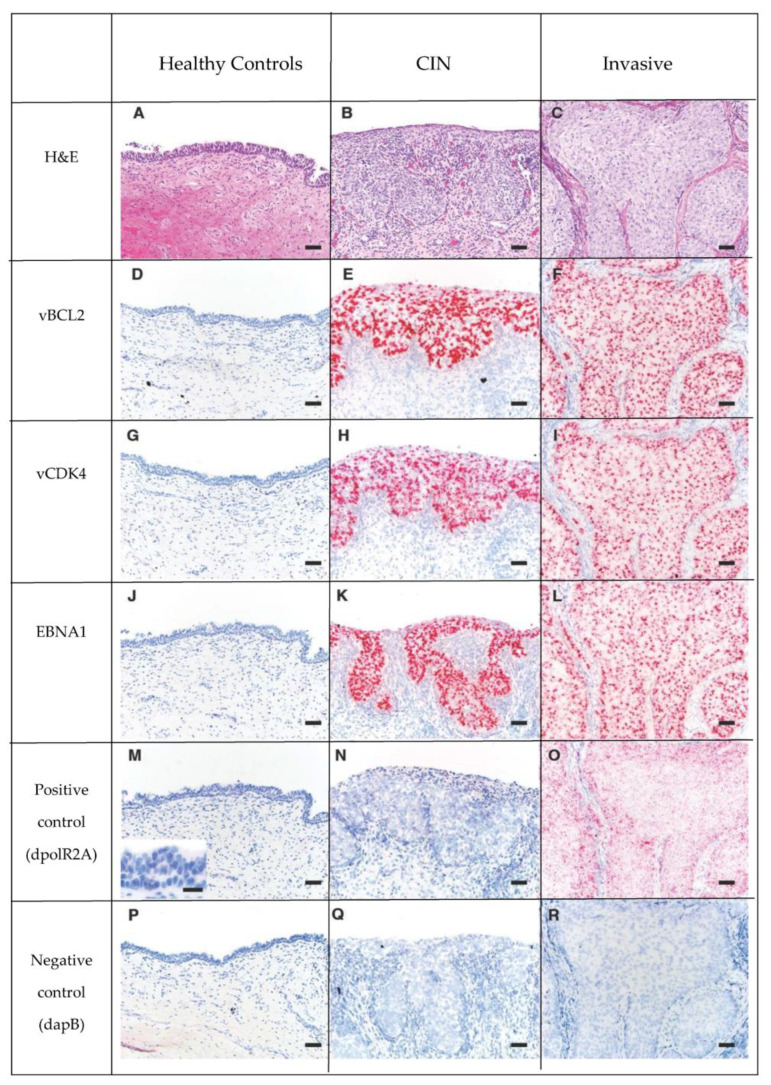
Series of H&E (Row 1: **A**–**C**) and RISH (Basescope^®^) labeled tissue (Rows 2 to 6: **D** through R), pink dots represent positive hybridization signaling. Rows 2, 3 and 4 are 3 OtHV1 RISH probes (Row 2- vBCL2, Row 3- vCDK4, Row 4-EBNA1). Row 5 is the positive control, California sea lion DNA-dependent RNA polymerase II (dpolR2A). Row 6 is the negative control dihydrodipicolinate reductase (dapB) of *Bacillus subtilis*. Note that there is no positive hybridization signal of the OtHV1 probes in the normal cervix (**D**,**G**,**J**), and there is a large amount of positive hybridization signaling in both the CIN (Column 2) and invasive (Column 3) lesions from cases with urogenital carcinoma. This shows that there is high OtHV1 mRNA expression in both CIN and invasive cervical tumors, but not in normal cervical epithelium. There was no positive hybridization signal in the negative control (Row 6) for control, CIN or invasive lesions.

The 16 cases with urogenital carcinoma (CIN and invasive) had significantly higher (*p* < 2 × 10^−16^) OtHV1 RISH binding for the five OtHV1 probes (EBNA1, vFLIP, vBCL2, vCDK4, and vEVE) compared to the control cervix (Figure 3). Positive hybridization signals of OtHV1 RISH probes were localized to the nucleus and to a lesser extent within the cytoplasm of the neoplastic epithelial cells. Quartile results for percent positive hybridization signal for all OtHV1 RISH (Basescope^®^) probes, positive and negative controls can be found in Supplemental Materials for control cases (Table S7), CIN (Table S8), and invasive lesions (Table S9). All control and cancer cases had no binding with the negative control RISH probe (dpol). There was no significant difference in polR2A hybridization signal among the healthy, CIN, or invasive lesions; thus, this cellular housekeeping positive control is an appropriate positive control for California sea lions with and without urogenital carcinoma.

**Figure 4:**
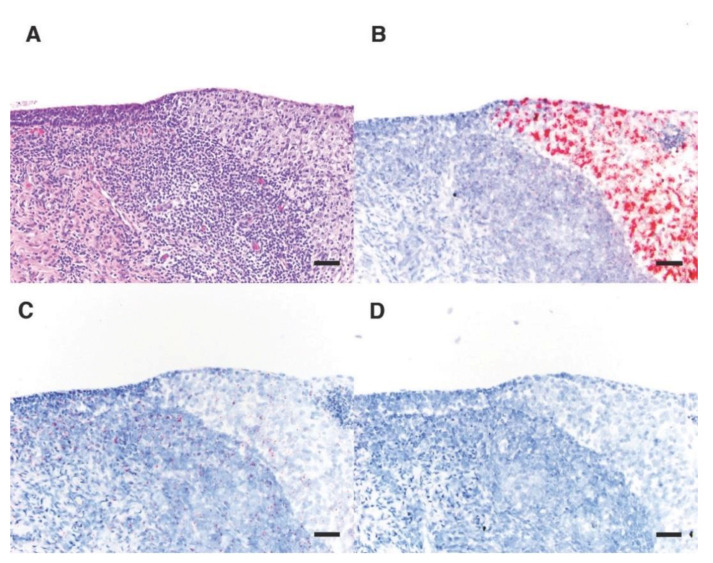
Histology section of the edge of a urogenital carcinoma lesion in the cervix of a California sea lion with normal epithelium (left side of images) transitioning into cervical intraepithelial carcinoma (CIN; right side of images) with inflammation in the underlying submucosa. Series shows (**A**) H&E and (**B**–**D**) RISH (Basescope^®^) labeled tissue (**B**) OtHV1vEVE; (**C**) host housekeeping gene DNA-dependent RNA polymerase II RISH positive control; (**D**) negative control dihydrodipicolinate reductase of *Bacillus subtilis*). On the RISH labeled tissues (**B**–**D**), pink dots represent positive hybridization signaling.

Most animals with urogenital carcinoma had diffuse lesions throughout the section of cervix sampled, with no normal epithelium. These cases had strong, evenly distributed, positive hybridization signal throughout the neoplastic tissue. However, one cancer case with both normal and neoplastic cervix within the examined sections had positive hybridization signal for all OtHV1 probes only in the neoplastic lesion and not in the surrounding normal epithelium (Figure 4). The positive control (pol2A) was evenly distributed throughout the normal and neoplastic epithelium.

## 4. Discussion

Infectious agents are associated with over 20% of cancers [13] and here we add OtHV1 and California sea lion urogenital carcinoma to that list. In this study, 100% of sea lions with urogenital carcinoma were infected with OtHV1. In line with previous studies, however, over a third of sea lions with normal cervical epithelium were also positive for OtHV1, but these animals had exceptionally low viral loads in their cervical tissue (median = 11,551 viral copies/ng DNA) and there was no viral gene expression detected in the healthy cervical epithelium. These findings support that OtHV1 is in the latent state in these infected sea lions that do not have urogenital carcinoma, similar to latently infected people with HPV, HHV4, or HHV8 that never developed associated malignancies.

This is in stark contrast with viral load and OtHV1 gene expression patterns in sea lions with urogenital carcinoma. Viral loads were very high in sea lions with urogenital carcinoma (median = 6,704,760 viral copies/ng DNA) and viral gene expression was significantly higher in cervical tumors but not in underlying stroma or normal cervical epithelium. This is profoundly apparent in the one case where both normal cervical epithelium and carcinoma in situ were observed in the same section (Figure 4). The very high expression of all five OtHV1 genes tested within the invasive portion of the cervix but not in the neighboring normal-appearing cervical epithelium support the hypothesis that OtHV1 plays a critical role in sea lion urogenital carcinoma development.

The model developed to predict a sea lion risk of having cancer based on the viral load provides a powerful clinical tool. With more than a third of the non-cancer sea lions (normal cervix) testing qPCR positive for OtHV1, this predictive model is a useful tool for interrupting sea lions with positive OtHV1 results and can help clarify if a positive OtHV1 result is a clinically significant finding indicative of cancer.

Phylogenetic analysis of OtHV1 showed it is most closely related to the herpesvirus family, gammaherpesviruses- several of which are known to have oncogenic potential. There was, however, an early branching of two pinniped herpesvirus (OtHV1 and OtHV4) and a dolphin herpesvirus (DeGHV1 associated with benign genital tumors) from the gammaherpesvirus subfamily. This early divergence indicates these three herpesviruses compose a novel genus of herpesviruses. The lack of a herpesvirus from a terrestrial mammal in the divergent clade may indicate that this group of herpesviruses co-evolved with marine mammals. In addition, there are several known viral oncogenes in HHV4 and HHV8 that alter signal transduction pathways, block apoptosis, and inactivate tumor suppressor pathways which contribute to their oncogenic potential [42,44]. Similar genes were identified in OtHV1, and further research is needed to better understand the roles these genes have in driving the development of this cancer.

Sea lions are top level coastal predators exposed to many of the same dietary contaminants and environmental stressors as humans. The now solidified link between OtHV1 and urogenital carcinoma in sea lions offers the opportunity to study naturally occurring herpesvirus-associated cancer in a novel species. This cancer can serve as a model for investigating viral/host interplay and give insight into basic cellular processes perturbed in these cancers in a naturally developing disease process. As in other virally induced cancers, there are likely several cofactors that contribute to the development of urogenital carcinoma. The oncogenic mechanisms employed by OtHV1 in urogenital carcinoma more realistically represent the pathophysiology of the virally induced cancer progression than traditional laboratory models because they are occurring in a natural host with an intact immune system and microenvironment representative of how this cancer develops under real-life conditions. Marine mammal rehabilitation centers along the west coast of the USA have access to these sick sea lions when they strand in end stage disease and euthanasia is the only humane option. Studying urogenital carcinoma in California sea lions can provide a valuable real-world model for virally induced cancer in a naturally occurring host [45].

## 5. Conclusions

Our findings conclude that OtHV1 plays an integral role in the pathogenesis of urogenital carcinoma in California sea lions. The significantly high viral load and exceptionally high viral gene expression within the cervical tumors in infected sea lions offer evidence that this cancer is virally induced. The low viral load and lack of viral gene expression in infected sea lions with normal cervical epithelium further provide support that if the virus is in a latent state there is no apparent effect on the cervical epithelium. In addition, composition of an OtHV1 draft genome identified several viral genes that are similar to previously identified oncogenes in other cancer-associated viruses. We hypothesize that, similar to other virally induced cancers, there are necessary cofactor(s) or triggering event(s) that cause OtHV1 to transition from a latent state to an activated state starting from the cervical epithelium down a pathway of cellular transformation and malignancy.

## Figures and Tables

**Table 1:** PCR and qPCR primers and probes used for OtHV1 and OtHV4 qPCR screening.

Primer/Probe Name	Primers (5′-3′)	Reference
OtGHV1_polF	CTTCGCATGGGTGGACTACT	This Study
OtGHV1_polR	TCATGCCTACTAGCAGCAGC	This Study
OtGHV1qPCRF	TCCCACGCTGTTTCGAATG	This Study
OtGHV1qPCRR	AGCTCCGAGTCGTGTACACAGTAT	This Study
OtGHV1_Probe	TCGCGCTCGCATCGGCA	This Study
OtGHV4F2	TCCACAATGATACTGGATGAAGA	Cortés-Hinojosa et al., 2016
OtGHV4R4	CTAGAATTGCACGACGCTGT	Cortés-Hinojosa et al., 2016
QPCR_OtGHV4F2	CTTCAACATTAGCTCCGGATT	Cortés-Hinojosa et al., 2016
QPCR_OtGHV4R2	CTTTACGCTTTGTTAGCCATGT	Cortés-Hinojosa et al., 2016
QPCR_OtGHV4probe2	AAAAAGCCATATATGTCAATCGCTACTATCAAA	Cortés-Hinojosa et al., 2016

## Data Availability

The data presented in this study are available on request from the corresponding author.

## Supplementary Materials

The following are available online at https://www.mdpi.com/2076-2615/11/2/491/s1: Methods S1: PCV and qPCR validation, Methods S2: PacBio and consensus PCR methods, Methods S3: RISH staining protocol, Methods S4: RISH quantification and analysis. Figure S1: H&E section of normal sea lion cervix. Figure S2: H&E section of urogenital carcinoma in sea lion cervix. Figure S3: H&E section of invasive urogenital carcinoma in sea lion cervix. Table S1: Herpesvirus species included in concatenated phylogenetic tree analysis. Table S2: Herpesvirus genes included in concatenated phylogenetic tree analysis. Table S3: Custom RISH probes. Table S4. Cervix histologic findings, OtHV1 qPCR results, and cause of death from California sea lions deemed controls used for RISH. Table S5: Cervix histologic findings, OtHV1 qPCR results, and cause of death from California sea lions deemed cancer cases used for RISH. Table S6: Quartile values of qPCR viral loads (OtHV1) from normal cervix and cervix with urogenital carcinoma. Table S7: Quartile values of OtHV1 RISH probes from sea lions with normal cervix. Table S8: Quartile values of OtHV1 RISH probes from sea lions with carcinoma intraepithelial neoplasia (carcinoma in situ) urogenital carcinoma. Table S9: Quartile values of OtHV1 RISH probes from sea lions invasion urogenital carcinoma.
