# Supplementary material for: Unlocking the Role of a Genital Herpesvirus, Otarine Herpesvirus 1, in California Sea Lion Cervical Cancer
Source: Animals (Basel). 2021 Feb 13;11(2):491. doi: 10.3390/ani11020491 (PMC7918579; doi:10.3390/ani11020491)
Supplement: Supplementary file 1 [file animals-11-00491-s001.pdf]

animals

MDPI

Supplemental Materials:

Methods S1. For PCR, OtHV1-specific PCR primers were designed (Table 1, OTHV1_polF, OTHV1_polR) and conditions were as follows: denaturation for 5 minutes at 94°C, followed by 45 cycles of denaturation at 94°C for 1 minute, annealing at 58°C, and extension at 72°C for 60 seconds, and an elongation step at 72°C for 7 minutes. PCR bands of appropriate size (344bp) were gel extracted with QIAquick Gel Extraction kit (Cat No. 28706; Qiagen Inc.), Sanger sequenced at University of Florida Interdisciplinary Center for Biotechnology Research (UF-ICBR) and sequence confirmed to be OtHV1 with 100% homology to the known OtHV1 polymerase sequence (GenBank accession # AF193617.1).

For qPCR standard curve generation, the 344bp OtHV1 dpol PCR product was run on a 1% agarose gel, extracted with QIAquick Gel Extraction kit (Cat No. 28706; Qiagen Inc.) and the amplicon was sequenced at the UF-ICBR using an ABI 3130 DNA sequencer (Life Technologies, Carlsbad, California, USA) and confirmed as OtHV1 dpol. DNA concentration was quantified using a NanoDrop 8000 spectrophotometer (Therma Scientific, Wilmington, Delware, USA). Ten-fold dilution series ranging from 10 to 10^7 copies per well were made with the PCR product and diluted with Tris-EDTA buffer. A 10^{-1}/slope was calculated for efficiency [1].

Sensitivity of the novel OtHV1-specific qPCR primers and probe (Table 1; OtHV1qPCRF, OtHV1qPCRR, OtHV1_Probe) were performed using a 10-fold dilution series of the OtHV1 dpol PCR product discussed above to estimate the analytical sensitivity (lower limit of detection for the assay). To test viral specificity, 10 diagnostic samples from California sea lion cervical tumors previously submitted to the authors' laboratory, which tested positive for OtHV1 on PCR and were confirmed to be OtHV1 by Sanger sequencing, were used. An additional 10 OtHV1 negative California sea lion cervix samples were included to assess species-specific negative controls. Four OtHV4 positive samples from Northern fur seal vaginal swabs, one OtHV3 positive sample from a California sea lion esophageal ulcer [2], and one herpesvirus positive sample from sea turtle cutaneous fibropapilloma were tested with the novel primer sets. All positive results were Sanger sequenced to confirm positive band sequences were OtHV1.

Each 20μL reaction was composed of 4μL DNA extract, 10μL qPCR Master mix (TaqMan® Fast Universal PCR Master Mix 2X, Applied Biosystems), 3μL of molecular grade water, and 1μL of each primer at a dilution of 18μM. All samples were run in duplicate with an internal positive control of 18S ribosomal universal eukaryote DNA primer/probe (VIC Probe, Applied Biosystems) on a 7500 Fast Real-Time PCR System (Applied Biosystems) using a standard Fast protocol with thermocycling conditions: 94°C for 20 seconds once, followed by 45 cycles at 94°C for 3 seconds and 60°C for 30 seconds. Reactions were run on 96-well polypropylene plates (Olympus Plastics, Genesee Scientific) and had 3 no template (molecular grade water) negative controls and 10 to 10^7 standard curves in triplicate. Data was analyzed using 7500 Fast Real-Time PCR System software, giving results as viral copies detected per nanogram of DNA in the reaction. To confirm no cross reaction between OtHV1 and OtHV4, all above mentioned samples were also tested for OtHV4 using PCR and qPCR with previously published primers, probe and conditions [3].

Methods S2: The PacBio RS II platform at the University of Florida Interdisciplinary Center for Biotechnology Research (UF-ICBR) was used for genomic sequencing [4]. The purpose was to generate the long insert libraries for long-read sequencing, in order to facilitate OtHV1 genome assembly. DNA quality was evaluated using the Agilent TapeStation with a Genomic Tape. The average DNA size had peak >60kb. Quantitation was performed by fluorescence (QUBIT, ThermoFisher). Eight micrograms of high MW genomic DNA were applied to a G-tube (Covaris, Inc.) using fragmentation conditions for 20kb. AMPure magnetic beads (Cat# A63881, Beckman Coulter) at 0.45:1.00 beads to sample ratio, were then used to clean the DNA before library

construction reactions. Large-insert (20 Kb) library construction was performed using 5 micrograms of G-tube fragmented DNA according to the PacBio protocol (P/N 100-286-000-07) with a few modifications. Briefly, SMRT bell adaptors were attached to the sample fragments in four basic steps: DNA damage repair, DNA end repair, ligation of adaptors, and exonuclease III/VII digestion. The final library yield was approximately 1.2 micrograms (~25% of the original mass). The final library was further size-selected on an Electrophoretic Lateral Fractionator (ELF, SageBioSciences), using a 0.75% Agarose (Native) Gel Cassettes v2 (Cat# ELD7510), specified for 0.8-18 kb fragments (3.61-hour run). This was done in order to maximize the average length for the "Reads of Insert" during sequencing. The final library was quantitated by fluorescence (QUBIT, ThermoFisher), and sized on an Agilent TapeStation (genomic tape). A total of ~200 ng of approximately 24 kb library fragments were recovered by pooling fractions corresponding to wells 1-3 from the ELF. This material was used to set up sequencing reactions in the PacBio RS II for two SMRT cells (Single Molecule Real Time) according to the manufacturer's protocol, using v3 SMRT cells and P6/C4 chemistry reagents, 6-hr movies. A 100pM on-plate loading concentration was used. Approximately 70,000 reads with an average polymerase read length of ~15kb were obtained per SMRT cell. Additionally, OtHV1 gap closure was attempted using novel primers (design based on generated draft genome) for conventional PCR using platinum Taq DNA Polymerase (Invitrogen, Carlsbad, CA, USA), GC RICH PCR System (Sigma-Aldrich, Atlanta, GA, USA) and TaKaRa Ex Taq® Hot Start Version (Takara Bio, Mountain View, CA, USA) and Sanger sequencing.

Methods S3. Eight serial FFPE sections from each case were cut onto four slides (two sections per slide). Slides were baked for 1 hour at 60˚C then dewaxed in xylene twice for 5 minutes, followed by dehydration in 100% ethyl alcohol twice for 2 minutes, and air-dried. RNAscope hydrogen peroxide (ACD, Hayward, California, cat #322381) was applied to each section and incubated for 5 minutes at room temperature, then slides were rinsed five times in double distilled water (ddH2O). Slides were placed in RNAscope 1X Target Retrieval Reagent (ACD, cat #322000) and incubated at a slow boil for 30 minutes. Slides were rinsed at room temperature in ddH2O, followed by 100% ethanol rinse and air-dried. A hydrophobic barrier was placed around each tissue section (ACD, ImmEdge TM hydrophobic Barrier Pen, cat #310018) and each section was treated with Protease III digestion buffer (ACD, Cat. #322381) for 30 minutes at 40˚C. Slides were washed in ddH20 and target or control probes applied and incubated for 2 hours at 40˚C. Basescope® custom probes targeting five viral genes (Table 4-1) included: OtHV1-LANA-like (4ZZ probe targeting 198-434), OtHV1-vFLIP (4ZZ probe targeting 223-504), OtHV1-vBCL2 (4ZZ probe targeting 3-240), OtHV1-vCDK4 (4ZZ probe targeting 3-855), OtHV1-vEVE (4ZZ probe targeting 39-261). To assess RNA quality, two additional custom positive control probes (aimed at cellular housekeeping genes) and one proprietary negative probe (aimed at a non-specific bacterial transcript) were used: polR2A (DNA-dependent RNA polymerase II, 3ZZ probe targeting sea lion, 555-705), and dapB (dihydrodipicolinate reductase, 3ZZ probe, ACD, cat #701011) of Bacillus subtilis. Following incubation, slides were washed in wash buffer (ACD, cat #310091) for 2 min at room temperature. Signal amplification reagents (AMP) 0-5 were applied as follows: AMP 0 for 30 minutes at 40˚C, AMP 1 for 15 minutes at 40˚C, AMP 2 for 30 minutes at 40˚C, AMP 3 for 30 minutes at 40˚C, AMP 4 for 15 minutes at 40˚C, and AMP 5 for 30 minutes at room temperature. Slides were rinsed for 2 minutes with wash buffer between amplification reagents. Positive signal was visualized using Fast RedTM dye incubated for 10 minutes at room temperature, rinsed with tap water, counterstained with Gill's hematoxylin, dried at room temperature, then coverslipped.

Methods S4. Using the IHC tool, the software was trained to recognize individual positive-stained pink areas by selecting ten representative punctate, well-defined pink dots from all probes in every case (healthy and cancer) and saved as a Reader User Model to analyze all images. The trained model was selected, and color space segmentation was used to define the positively stained areas. The image type was then converted to 8-bit, and a threshold of 200 was set for all images. Control cervix and vagina had a thinner simple columnar epithelial layer compared to neoplastic lesions. To ensure similar sized areas were being evaluated in normal epithelium, the percent positive

hybridization signal was measured over three 40x fields and averaged. Therefore, for controls, three representative areas between 160,870 to 1,300,000 per 40x field of normal epithelium were selected and added, totaling a region of interest for healthy animals ranging from 482,610 to 3,900,000. In CIN and invasive lesions an area between 523,000 to 3,200,000 was selected within one 40x field and percent positive hybridization signal was calculated as described above.

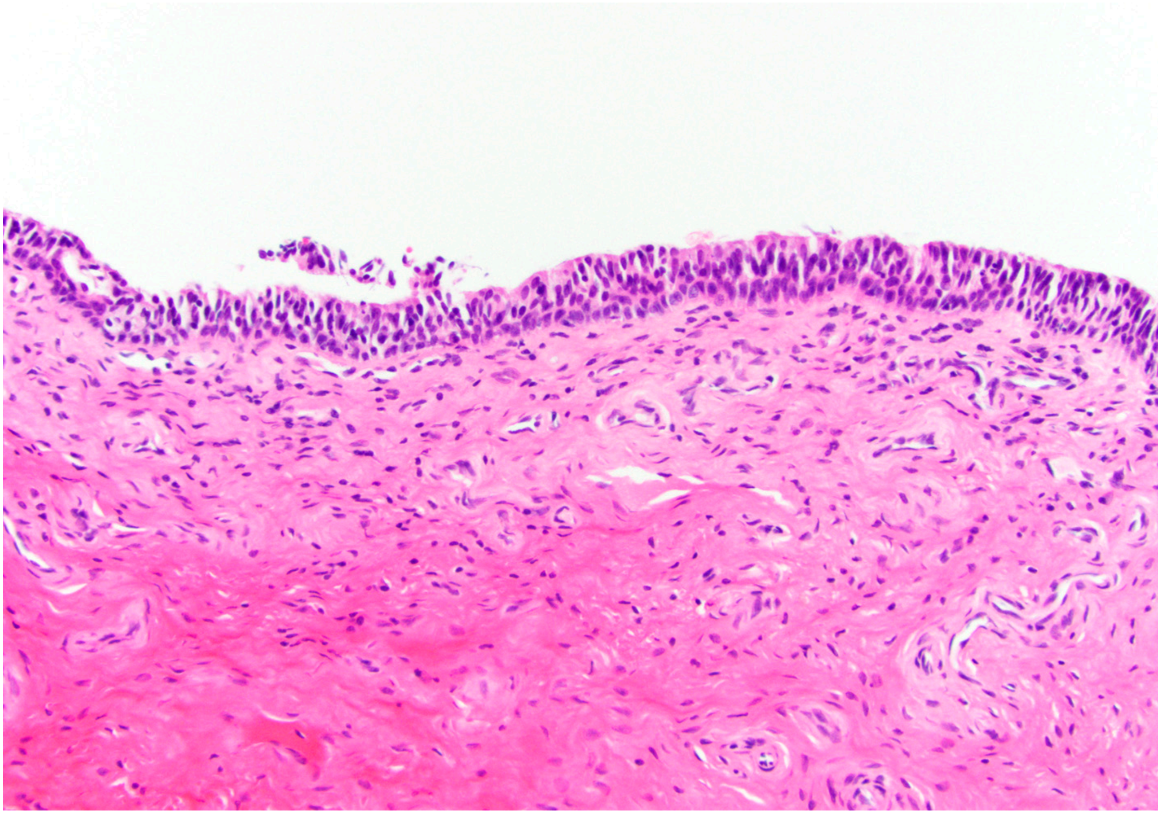

Figure S1. H&E. Histology sections of normal cervix from an adult California sea lion (*Zalophus californianus*) composed of pseudostratified columnar epithelium and underlying stroma.

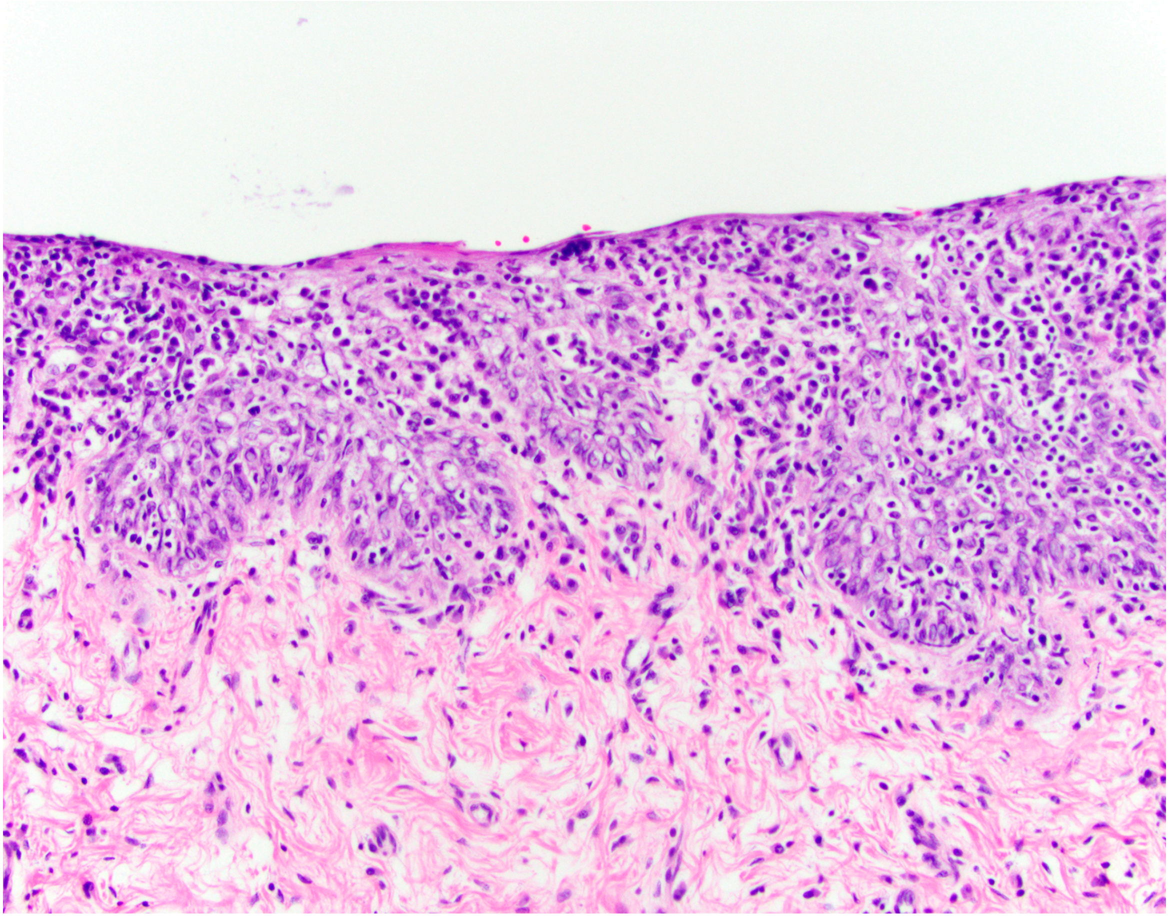

Figure S2. H&E. Histology sections of urogenital carcinoma showing cervical intraepithelial neoplasia (CIN) in an adult California sea lion (*Zalophus californianus*) composed of moderate to markedly dysplastic cervical epithelium with atypical parabasal cell proliferation extending from one third to the entire thickness of the epithelium. The underlying submucosa has lymphocytic inflammation.

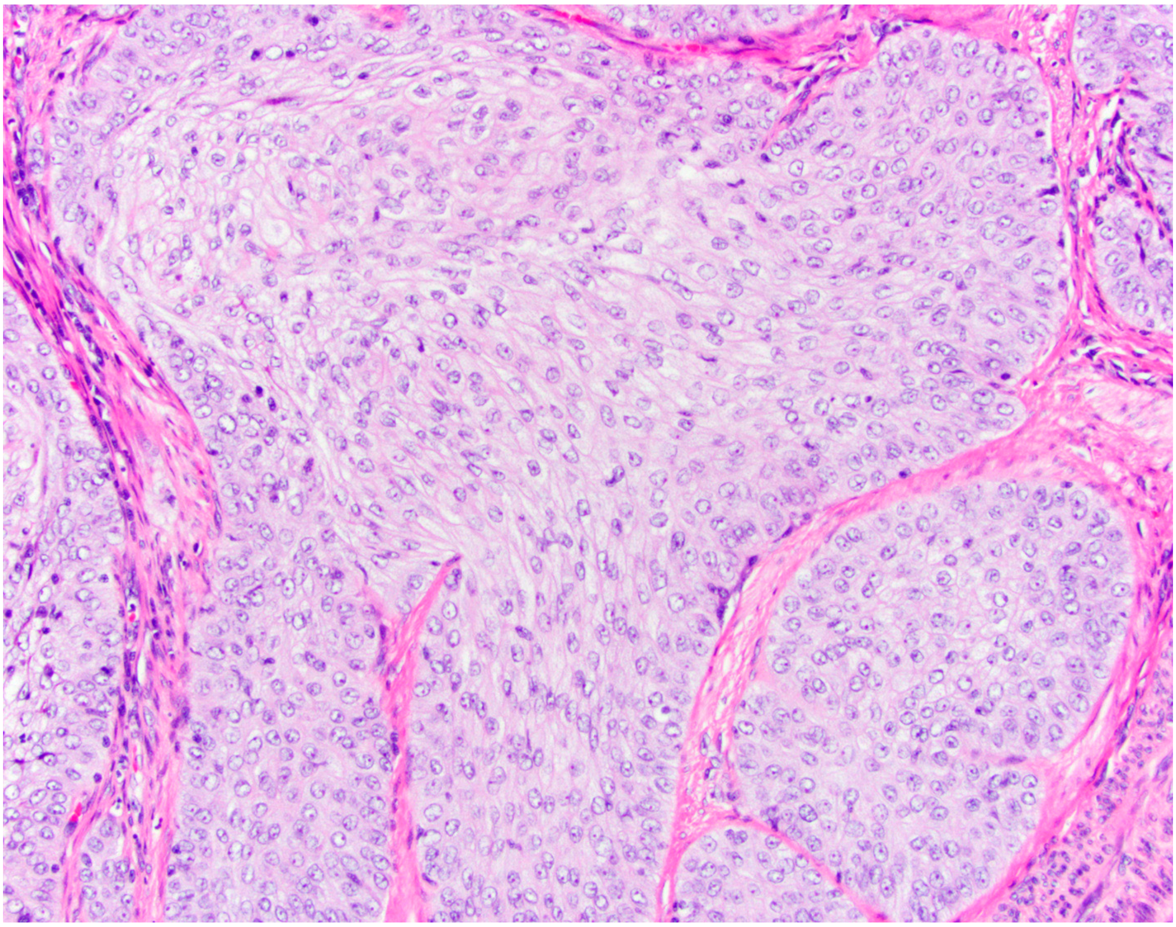

Figure S3. H&E. Histology sections of invasive urogenital carcinoma (Case 1094-1) from the cervix of an adult California sea lion (*Zalophus californianus*). Here, large islands of neoplastic cells are deep within the submucosa and lack a basement membrane (infiltration).

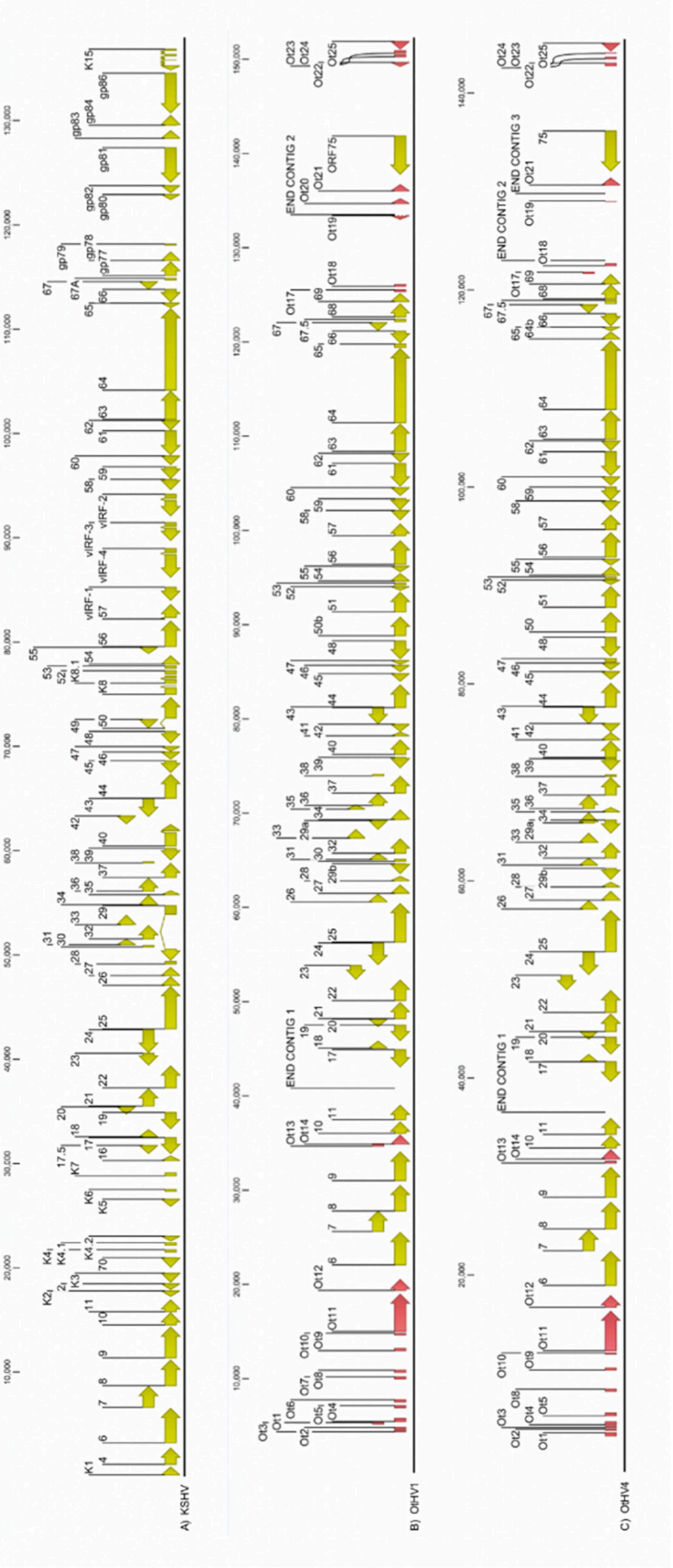

Figure S4. Annotated genomes of A) HHV8 (GenBank accession number: NC_009333); B) OtGHV1 (GenBank accession numbers: Contig1=MN334559, Contig2= MN334560, Contig3= MN334561); and C) OtGHV4 (GenBank number: Contig1= MN545486, Contig2= MN545487, Contig3= MN545488, Contig4= MN545489). The genes in yellow indicate core herpesvirus genes in OtGHV1 and OtGHV4 that are colinear with HHV8. The genes in red in B and C are open reading frames that had no identifiable putative protein and were annotated with a prefix of "Ot", for Otarine, and a number corresponding to gene order starting at the 5′ end of the genome. In OtGHV1 (B) and OtGHV4 (C) "END CONTIG" indicates a gap in the assembly. Contig order is displayed collinearly with HHV8 for display purposes.

Table S1. List of herpesvirus species included in concatenated phylogenetic tree analysis.

Virus name	Abbreviation	Accession #	Subfamily	Genus
Human alphaherpesvirus 3	Human HV3	NC_001348.1	Alpha	*Simplex*
Suid alphaherpesvirus 1	Suid AHV1	NC_006151.1	Alpha	*Varicellovirus*
Elephantid betaherpesvirus 1	Elephantid BHV1	NC_020474.2	Beta	*Proboscivirus*
Human betaherpesvirus 7	Human HV7	NC_001716.2	Beta	*Roseolovirus*
Callitrichine herpesvirus 3	Callitrichine HV3	NC_004367.1	Gamma	*Lymphocryptovirus*
Human gammaherpesvirus 4	Human HV4	NC_007605.1	Gamma	*Lymphocryptovirus*
Macacine gammaherpesvirus 4	Macacine GHV4	NC_006146.1	Gamma	*Lymphocryptovirus*
Alcelaphine gammaherpesvirus 1	Alcelaphine GHV1	NC_002531.1	Gamma	*Macavirus*
Bovine gammaherpesvirus 6	Bovine GHV6	NC_024303	Gamma	*Macavirus*
Ovine gammaherpesvirus 2	Ovine GHV2	NC_007646.1	Gamma	*Macavirus*
Porcine lymphotropic herpesvirus 2	Porcine lymphotrophic HV2	AA0012350	Gamma	*Macavirus*
Equid gammaherpesvirus 2	Equid GHV2	NC_001650.2	Gamma	*Percavirus*
Equid gammaherpesvirus 5	Equid GHV5	NC_026421.1	Gamma	*Percavirus*
Ateline gammaherpesvirus 3	Ateline GHV3	NC_001987.1	Gamma	*Rhadinovirus*
Bovine gammaherpesvirus 4	Bovine GHV4	NC_002665.1	Gamma	*Rhadinovirus*
Ceropithecine herpesvirus 17	Ceropithecine HV17	NC_003401	Gamma	*Rhadinovirus*
Cricetid gammaherpesvirus 2	Cricetid GHV2	NC_015049.1	Gamma	*Rhadinovirus*
Human gammaherpesvirus 8	Human HV8	NC_009333.1	Gamma	*Rhadinovirus*
Murid gammaherpesvirus 4	Murid HV4	NC_001826	Gamma	*Rhadinovirus*
Retroperitoneal fibromatosis-associated herpesvirus	Macaque RFHVMn	KF703446	Gamma	*Rhadinovirus*
Saimiriine gammaherpesvirus 2	Saimiriine GHV2	NC_001350.1	Gamma	*Rhadinovirus*
Delphinid gammaherpesvirus 1	Delphinid GHV1	NC_035117.1	Gamma	*Unclassified*
Felis catus gammaherpesvirus 1	Felis catus GHV1	NC_028099.1	Gamma	*Unclassified*
Harp seal herpesvirus	Harp Seal HV	KP136799.1	Gamma	*Unclassified*
Myotis gammaherpesvirus 8	Myotis GHV8	NC_029255	Gamma	*Unclassified*
Otarine herpesvirus 1	OtHV1	submitted	Unclassified	*Unclassified*
Otarine herpesvirus 4	OtHV4	submitted	Unclassified	*Unclassified*

Table S2. List of herpesvirus genes GenBank accession numbers included in concatenated phylogenetic tree analysis.

Virus name	Polymerase (ORF 9)	Terminase (ORF 7)	Glycop B (ORF 8)	Major capsid (ORF 25)
Human alphaherpesvirus 3	NP_040151	NP_040153	NP_040154	NP_040163.1
Suid alphaherpesvirus 1	YP_068333	YP_068331	YP_068330	YP_068356.1
Elephantid betaherpesvirus 1	YP_007969814	YP_007969816	YP_007969815	YP_007969788.1
Human betaherpesvirus 7	YP_073778	YP_073780	YP_073779	YP_073799.1
Callitrichine herpesvirus 3	NP_733857	NP_733855	NP_733856	NP_733870
Human gammaherpesvirus 4	YP_401712	YP_401715	YP_401713	YP_401697
Macacine gammaherpesvirus 4	YP_068007	YP_068010	YP_068009	YP_067994
Alcelaphine gammaherpesvirus 1	NP_065512	NP_065510	NP_065511	NP_065524
Bovine gammaherpesvirus 6	YP_009041990	YP_009041988	YP_009041989	YP_009042004
Ovine gammaherpesvirus 2	YP_438136	YP_438134	YP_438135	YP_438149
Porcine lymphotropic herpesvirus 2	AAO12282	AAO12280	AAO12281	AAO12367
Equid gammaherpesvirus 2	AIU39456	AIU39454	AIU39455	NP_042621
Equid gammaherpesvirus 5	YP_009118399.1	YP_009118397.1	YP_009118398.1	YP_009118415.1
Ateline gammaherpesvirus 3	NP_047983	NP_047981	NP_047982	NP_047996
Bovine gammaherpesvirus 4	NP_076501	NP_076499	NP_076500	NP_076517
Ceropithecine herpesvirus 17	NP_570750.1	NP_570748.1	NP_570749.1	NP_570765.1
Cricetid gammaherpesvirus 2	YP_004207849.1	YP_004207847	YP_004207848.1	YP_004207861.1
Human gammaherpesvirus 8	YP_001129355	YP_001129353.1	YP_001129354	YP_001129378
Murid gammaherpesvirus 4	NP_044849	NP_044847	NP_044848	NP_044863
Retroperitoneal fibromatosis-associated herpesvirus	AGY30688	AGY30686	AGY30687	AGY30708
Saimiriine gammaherpesvirus 2	NP_040211	NP_040209	NP_040210	NP_040227
Dephinid gammaherpesvirus 1	YP_009388514.1	YP_009388512.1	YP_009388513.1	YP_009388526.1
Felis catus gammaherpesvirus 1	YP_009173887.1	YP_009173885.1	YP_009173886.1	YP_009173900.1
Harp seal herpesvirus	AJG42938.1	AJG42936.1	AJG42937.1	AJG42951.1
Myotis gammaherpesvirus 8	YP_009229846	YP_009229844	YP_009229845	YP_009229859
Otarine herpesvirus 1	submitted	submitted	submitted	submitted
Otarine herpesvirus 4	submitted	submitted	submitted	submitted

Table S3. Custom RISH probes (Basescope®) targeting 5 viral genes, a positive control and a negative control (Column 1). Proposed homologous gene (Column 2), mechanism of action (MOA) for known genes (Column 3) and number (#) zz pairs (Column 4) provided.

Probe name	Homologous gene	Proposed MOA for genes	# ZZ pairs
OtGHV1-EBNA1	Epstein-Barr Nuclear Antigen 1	Anti-apoptotic, induces cellular Proliferation; causes chromosomal instability	4ZZ
OtGHV1-vFLIP	Viral Fas-associated death domain-like interleukin-1β-converting enzyme-inhibitory protein	Anti-apoptotic; binds Caspase 8 and blocks death receptor-induce apoptosis	4ZZ
OtGHV1-vBCL2	Viral B-cell lymphoma 2	Anti-apoptotic and immune system evasion	4ZZ
OtGHV1-vCDK4	Viral cyclin-dependent kinase 4	Induces cellular proliferation	4ZZ
OtGHV1-vEVE	Viral endogenous viral element	Suspected retroviral element inserted in viral and host genome	4ZZ
Zc-polR2A	*Zalophus californianus* DNA-dependent RNA polymerase II	Catalyzes the transcription of DNA to mRNA and most snRNA and microRNA	3ZZ
dapB	*Bacillus subtilis* dihydrodipicolinate reductase	Component of the lysine biosynthetic pathway in bacteria and higher plants	3ZZ

Table S4. Cervix histologic findings and OtHV1 qPCR results from California sea lions deemed controls (non-cancer cases; n=9) and used for OtHV1 RNA *in situ* hybridization investigation. Note that four of the control cases had normal cervix on histologic evaluation but were positive for OtHV1.

Patient ID	Collection date	Histology description	OtHV1 qPCR results
CSL 11220	7/9/14	Normal	Positive
CSL 12902	3/4/16	Normal	Negative
CSL 13002	4/6/16	Normal	Negative
CSL 13383	6/19/17	Normal	Negative
CSL 13399	7/5/17	Normal	Positive
CSL 13453	7/26/17	Normal	Negative
CSL 13483	8/9/17	Normal	Negative
CSL 13491	8/9/17	Normal	Positive
CSL 13467	8/14/17	Normal	Positive

Table S5. Cervix histologic findings and OtHV1 qPCR results from California sea lions deemed cancer cases (n=16) and used for OtHV1 RNA *in situ* hybridization investigation. Note that all the cancer cases had evidence of either carcinoma *in situ* (n=7) or invasive carcinoma (n=9) on histologic evaluation and all were positive for OtHV1 (n=16).

Patient ID	Collection date	Histology description	OtGHV1 qPCR results
CSL 10337	6/19/12	Carcinoma *in situ*	Positive
CSL 10462	11/8/12	Carcinoma *in situ*	Positive
CSL 10611	4/9/13	Carcinoma *in situ*	Positive
CSL 10675	7/24/13	Carcinoma *in situ*	Positive
CSL 10707	8/27/13	Carcinoma *in situ*	Positive
CSL 10778	12/17/13	Carcinoma *in situ*	Positive
CSL 13473	8/1/17	Carcinoma *in situ*	Positive
CSL 13479	8/14/17	Invasive	Positive
CSL 10240	11/13/11	Invasive	Positive
CSL 10273	1/29/12	Invasive	Positive
CSL 10482	12/20/12	Invasive	Positive
CSL 10689	8/2/13	Invasive	Positive
CSL 12597	7/2/15	Invasive	Positive
CSL 13337	5/19/17	Invasive	Positive
CSL 13325	5/08/17	Invasive	Positive
CSL 13385	6/22/17	Invasive	Positive

Table S6. Quartile values for viral copies per nanogram of DNA (qPCR) for OtHV1 positive normal cervix (control) and cervix with urogenital carcinoma (cancer).

**Quartile values**	**Control (n=59)**	**Cancer (n=95)**
Minimum value	1,061	9,702
First quartile	3,175	1,284,055
Median value	11,551	6,704,760
Third quartile	64,166	14,472,650
Maximum value	15,158,300	42,421,500

Table S7. Quartile results for percent positive hybridization signal in the 9 control cases for all OtHV1 RISH (Basescope®) probes and positive controls.

Healthy (n=9)	OtHV1 Basescope® Probes					Positive controls
	EBNA1	vFLIP	vBCL2	vCDK4	vEVE	polR2A
Minimum	0.000	0.000	0.000	0.000	0.000	0.075
First quartile	0.000	0.001	0.000	0.000	0.002	0.357
Median	0.001	0.002	0.001	0.001	0.052	0.531
Third quartile	0.003	0.010	0.006	0.001	0.079	0.648
Maximum	0.023	0.139	0.052	0.005	0.259	1.433

Table S8. Quartile results for percent positive hybridization signal in the 7 urogenital carcinoma intraepithelial neoplasia (CIN) urogenital carcinoma lesions for all OtHV1 RISH (Basescope®) probes and positive control.

CIN (n=7)	OtHV1 Basescope® Probes					Positive controls
	EBNA1	vFLIP	vBCL2	vCDK4	vEVE	polR2A
Minimum	8.195	21.430	40.147	23.487	24.202	0.321
First quartile	39.122	47.746	49.316	45.246	46.039	0.449
Median	49.551	54.320	54.191	54.744	53.979	0.596
Third quartile	56.197	64.469	58.212	60.065	63.894	1.609
Maximum	69.639	71.489	79.967	75.510	75.738	2.232

Table S9. Quartile results for percent positive hybridization signal in the 9 invasive urogenital carcinoma lesions for all OtHV1 RISH (Basescope®) probes and positive control.

Invasive (n=9)	OtHV1 Basescope® Probes					Positive controls
	EBNA1	vFLIP	vBCL2	vCDK4	vEVE	polR2A
Minimum	22.034	24.484	23.740	17.319	32.263	0.061
First quartile	31.238	31.476	31.527	29.839	35.189	0.304
Median	37.268	43.612	34.984	39.831	41.394	0.661
Third quartile	47.499	50.575	49.084	46.083	53.586	1.034
Maximum	73.571	61.410	66.312	64.768	70.415	1.855

References

1. Bustin, S.A.; Benes, V.; Garson, J.A.; Hellemans, J.; Huggett, J.; Kubista, M.; Mueller, R.; Nolan, T.; Pfaffl, M.W.; Shipley, G.L.; et al. The MIQE guidelines: Minimum information for publication of quantitative real-time PCR Experiments. Clin. Chem. 2009, 55, 611–622, doi:10.1373/clinchem.2008.112797.
2. 2Venn-Watson, S.; Benham, C.; Gulland, F.; Smith, C.R.; Leger, J.S.; Yochem, P.K.; Nollens, H.; Blas-Machado, U.; Saliki, J.T.; Colegrove, K.M.; et al. Clinical relevance of novel Otarine herpesvirus-3 in California sea lions (Zalophus californianus): lymphoma, esophageal ulcers, and strandings. Veter. Res. 2012, 43, 85, doi:10.1186/1297-9716-43-85.
3. Cortés-Hinojosa, G.; Gulland, F.M.D.; Delong, R.; Gelatt, T.; Archer, L.; Wellehan, J.J.F.X. A novel gammaherpesvirus in northern fur seals (Callorhinus ursinus) is closely related to the california sea lion (Zalophus californianus) carcinoma-associated otarine herpesvirus-1. J. Wildl. Dis. 2016, 52, 88–95, doi:10.7589/2015-03-060.
4. English, A.C.; Richards, S.; Han, Y.; Wang, M.; Vee, V.; Qu, J.; Qin, X.; Muzny, D.M.; Reid, J.G.; Worley, K.C.; et al. Mind the Gap: Upgrading Genomes with Pacific Biosciences RS Long-Read Sequencing Technology. PLoS ONE 2012, 7, e47768, doi:10.1371/journal.pone.0047768.
